# Acute burst fracture in Kummell’s disease with acute onset neurological deficit: a case report on role of spinal stability and technical notes on “pivot ligamentotaxis”

**DOI:** 10.1186/s12893-019-0511-y

**Published:** 2019-05-14

**Authors:** Hyeun Sung Kim, Ravindra Singh, Nitin Maruti Adsul, Sung Woon Oh, Jung Hoon Noh, Jun Hwan Park, I. L. Tae Jang, Seong Hoon Oh

**Affiliations:** 1 Department of Neurosurgery, Nanoori Hospital, Seoul, 731, Eonju-ro, Gangnam-gu, Seoul, Republic of Korea ZIP-06048; 2 Medical School University of Debrecen, Debrecen, Hungary; 3 Department of Neurosurgery, Nanoori Incheon Hospital, Incheon, Republic of Korea

**Keywords:** Kummell’s disease, Neurological deficit, Burst fracture, Polymethylmethacrylate augmented, posterior compressed, short segment percutaneous pedicle screw fixation (PA-SSPPF), Pivot ligamentotaxis

## Abstract

**Background:**

Kummell’s Disease has insidious progression. Neurological deficit is usually slow in onset and progression and only few cases of acute neurological deficit have been reported. We came across a case of Kummell’s disease which progressed to burst fracture, developed neurological deficit within two weeks. We managed patient with “pivot ligamentotaxis” and Polymethylmethacrylate augmented, posterior compressed, short segment percutaneous pedicle screw fixation.

**Case presentation:**

Eighty-three years old woman following fall was on conservative management at another hospital. She had no neurological deficit. A week later her back pain aggravated and two weeks later developed bilateral buttock pain, bilateral lower limb weakness and diminished sensation in the sacral area.

Radiological investigations (X-rays, Magnetic resonance imaging and Computed tomography) showed L1 vertebral body fracture with vacuum cleft and fracture fragment retropulsed into the spinal canal.

A diagnosis of Kummell’s disease with burst fracture of L1 vertebra & neurological deficit was made.

Patient was managed with Polymethylmethacrylate augmented, posterior compressed, short segment percutaneous pedicle screw fixation. The reduction of the retropulsed fragment was achieved by virtue of “Pivot ligamentotaxis”. The patient got relieved of the symptoms (Preoperative VAS 8 and postoperative VAS 3) and was allowed brace assisted ambulation on first postoperative day.

**Conclusion:**

This study reports acute occurrence of the burst fracture in unstable vertebra inflicted by Kummell’s disease and role of spinal stability in recovery. We achieved closed reduction of the fracture fragments and relief of the cord compression by posterior compression with “pivot ligamentotaxis”.

## Background

The osteoporotic vertebral compression fractures and Kummell’s disease (KD) have become common [1, 2]. Kummell’s disease is a disease caused by osteonecrosis and can lead to severe vertebral collapse, which can lead to severe pain and may progress to neurological deficit [1, 3]. Neurological deficit in KD usually progresses slowly. Two cases of acute neurological deficit in KD have been reported [4, 5]. We report a case with acute occurrence and progression of neurological deficit in early phase of KD associated with burst fracture of the vertebral body which was managed by reduction using “pivot ligamentotaxis” and Polymethylmethacrylate augmented posterior compressive, short segment percutaneous pedicle screw fixation. The neurological status was restored after stabilization.

## Case presentation

### Case History

Eighty-three years old woman following a fall was put on conservative management at another hospital. Initially her physical examination was mostly normal other than spinal tenderness and she had no neurological (Sensory or motor) deficit. A week later she started having severe back pain and two weeks later she also developed bilateral buttocks pain, bilateral lower limb weakness and had diminished sensation in the sacral area.

### Physical Examination

Revealed severe tenderness at thoracolumbar junction and neurologic examination demonstrated grade 4/5 strength in the lower extremities. Sacral sensation decreased, bladder and bowel function was intact.

### Investigations

**Fig. 1:**
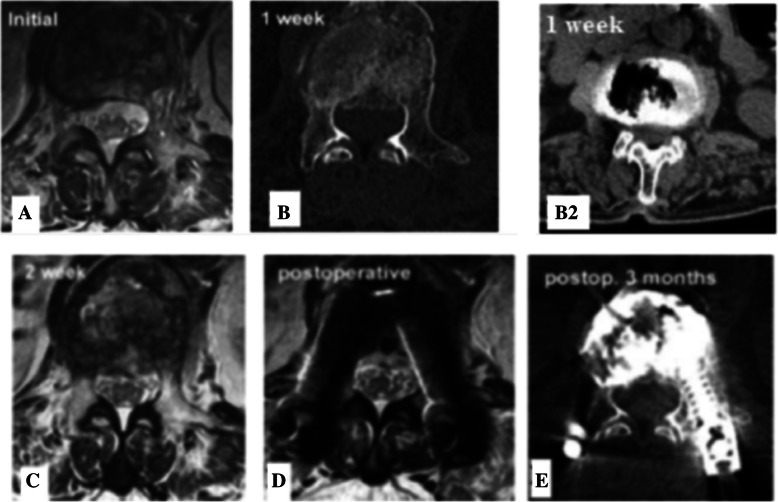
Axial MRI & CT Showing Rapid Progression and Resolution of Burst Fracture and Compression in a case of Kummell’s Disease. **a** Initial MRI, **b1** MRI at 1 week, **b2** CT at 1 week, **c** MRI at 2 week, **d** Post-operative MRI, **e** Postoperative CT 3 months

**Fig. 2:**
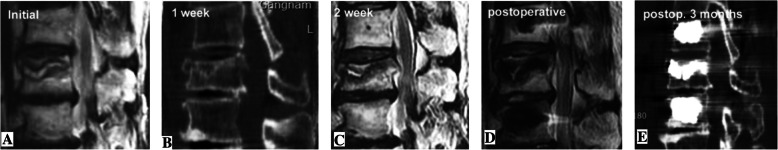
Sagittal MRI Showing Rapid Progression and Resolution of Burst Fracture and Compression in a case of Kummell’s Disease. **a** Initial MRI, **b** MRI at 1 week, **c** MRI at 2 week

Initial magnetic resonance imaging (MRI) revealed vertebral compression fracture of L1 body without burst and no canal compromise (Figs. 1a & 2a). One week later the MRI and computed tomography (CT) revealed compression fracture with intravertebral cleft and beginning of the burst of the vertebral body. A fragment was retropulsed into the canal (Figs. 1b, b2, and 2b). An MRI two weeks from initial fall shows aggravation of the burst fracture and thecal sac compression (Figs. 1c & 2c). A *diagnosis* of Kummell’s disease at L1 vertebra with burst fracture of the body with spinal canal compromise and neurological deficit was made.

**Fig. 3:**
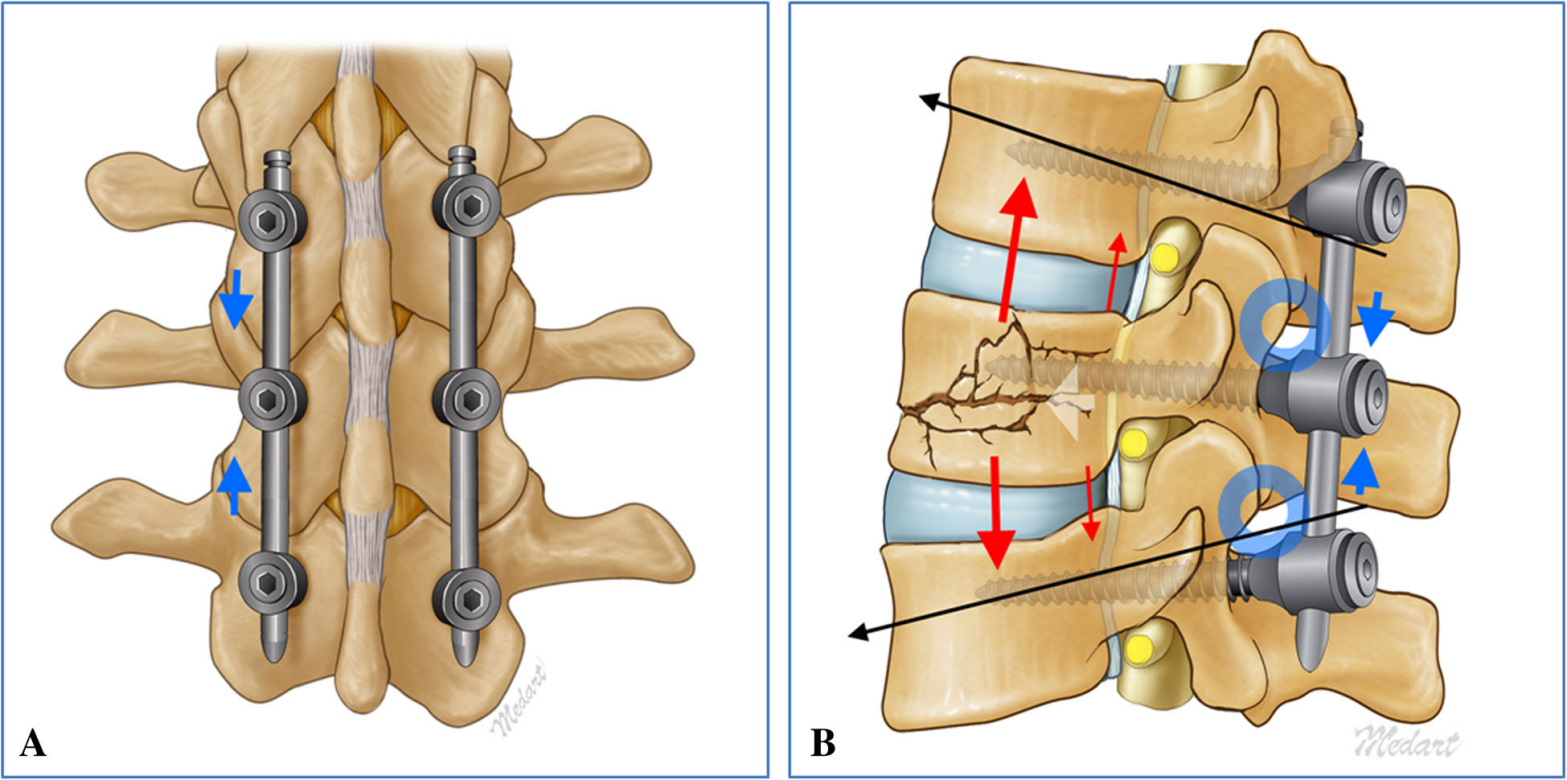
**a** Coronal and **b** Sagittal images showing the mechanism of “Pivot ligamentotaxis”

### Treatment

Polymethylmethacrylate (PMMA) augmented posterior compressive, short segment percutaneous pedicle screw (PA-SSPPF) fixation was done at T12, L1 and L2 vertebrae levels. Postoperatively the height of L1 body was restored and thecal compression disappeared (Figs. 1d & 2d). The patient experienced significant pain relief (Preoperative VAS 8 and postoperative VAS 3) and could ambulate with a brace on first postoperative day. Three months later the vertebral body height, stability was well preserved and patient had no neurological deficit (Figs. 1e & 2e).

### Pivot Ligamentotaxis

Is a novel concept in minimally invasive spine surgery (MISS). In our case the left pedicle screws of the T12, L1 & L2 were inserted first followed by the right screw of the L1 vertebra. Using the inserter the rod was slide through the pedicle screws on right side. Rod blockers of T12 pedicle screw was tightened first then T12 & L1 pedicle screws compressed and with the help of the compressor placed horizontally a cranially distractive force applied using left L1 pedicle screw as pivot points and the rod blockers of the right L1 pedicle screw tightened. This maneuver is repeated with of L1 and L2 where a caudally directed distractive force was applied using L1 pedicle as pivot. The cumulative result of this maneuver was ligamentotaxis and reduction of the retropulsed fragment Figs. 1d, 2d, and 3a & b). This was followed by insertion of remaining two contralateral pedicle screws and fixation. As described earlier all the screws were PMMA augmented inserted using minimally invasive screw insertion technique.

## Discussion and conclusion

Not many cases of Kummell’s disease associated with neurological deficit have been reported and a few reports of KD patients who presented with early onset and acute neurological deficit [4, 5]. However, we could not find any study or case report which mentions acutely occurring burst fracture of the involved vertebra as inciting event for the neurological compromise.

The dynamic instability at the vertebral body fracture adversely affects the fracture union, promotes vascular compromise which may result into psuedarthrosis and osteonecrosis [1, 2, 6]. Thus spinal instability has a role to play in the progression of the Kummell’s disease a fact supported by our study. Kim et al. had proposed Spinal Instability Predictive Score (SIPS) for KD patients with vertebroplasty which scores and grades instability [2]. There is no such score to predict the progression from osteoporotic vertebral fracture to the Kummel’s disease.

Conservative treatment for KD includes bed rest, brace, pain relief and osteoanabolic therapy (e.g. Bisphosphonates and Teriperatide) [1, 3].

Various surgical modalities are summarized in comprehensive review by Formica M et al. [1]. Laminectomy, and hematoma removal mentioned for neurological deficit associated with intraspinal hematoma [4, 5]. Bone cement augmented percutaneous pedicle screw stabilization also has good results in Kummell’s disease [7, 8].

We performed PMMA augmented, posterior compressed, percutaneous short segment pedicle screw fixation (PA-SSPPF) involving T12, L1, L2 vertebrae. The patient improved rapidly in postoperative period with the magnetic resonance imaging (MRI) showing resolution of canal compromise. Fast recovery of the patient can be attributed to the spinal stability achieved following fixation. We could reduce the fracture fragments into place and relieve thecal sac compression by applying posterior compression. This reduction was possible due to pivoting around the contralateral pedicle and we name this phenomenon as “pivot ligamentotaxis”. The pivot ligamentotaxis converts compression applied posterior into ligamentotaxis forces in the ligaments anterior to the facet joints e.g. posterior longitudinal ligaments (PLL) and anterior longitudinal ligaments (ALL) by virtue of pivoting around the facet joints.

This study shows that the neurological status can deteriorate rapidly following burst fracture of the involved vertebral body. The expeditious deterioration and prompt recovery of the patient affirms the role of spinal stability in the pathogenesis of burst fracture in KD and neurological deficit. “Pivot ligamentotaxis” allowed us to achieve closed reduction of fracture and decompression of the thecal sac. However, a study with larger sample and longer follow up would be required to derive a significant conclusion. In addition, the concept of “pivot ligamentotaxis” needs further evaluation with biomechanical studies. Also there is need for a predictive scoring system which could predict the possibility of progression of vertebral compression fracture to Kummell’s disease and occurrence of burst fracture at the earliest stage, so that appropriate measures could be taken.

## Abbreviations

ALL: Anterior longitudinal ligament
CT: Computed tomography
KD: Kummell’s disease
MISS: Minimally invasive spine surgery
MRI: Magnetic resonance imaging
PA-SSPPF: Polymethylmethacrylate augmented, posterior compressed, short segment percutaneous pedicle screw fixation
PLL: Posterior longitudinal ligament
PMMA: Polymethylmethacrylate
SIPS: Spinal instability predictive score
VAS: Visual analogue score
